# Chitosan non-particulate vaccine delivery systems

**DOI:** 10.3389/jpps.2024.12921

**Published:** 2024-07-24

**Authors:** Rasim Masimov, Ellen K. Wasan

**Affiliations:** College of Pharmacy and Nutrition, University of Saskatchewan, Saskatoon, SK, Canada

**Keywords:** non-particulate delivery systems, thermosensitive hydrogels, microneedles, conjugate vaccines, chitosan coating, vaccine, mucosal delivery

## Abstract

Chitosan is an extensively used polymer for drug delivery applications in particulate and non-particulate carriers. Chitosan-based particulate, nano-, and microparticle, carriers have been the most extensively studied for the delivery of therapeutics and vaccines. However, chitosan has also been used in vaccine applications for its adjuvant properties in various hydrogels or as a carrier coating material. The focus of this review will be on the usage of chitosan as a vaccine adjuvant based on its intrinsic immunogenicity; the various forms of chitosan-based non-particulate delivery systems such as thermosensitive hydrogels, microneedles, and conjugates; and the advantages of its role as a coating material for vaccine carriers.

## Introduction

Vaccines may be grouped under three generations: whole/inactivated vaccines, acellular/subunit vaccines, and nucleic acid-based vaccines as first, second, and third-generation vaccines, respectively [1]. Whole/inactivated vaccines are prepared by treating the disease-causing pathogens with inactivating chemicals to disable their ability to replicate and cause infection. Due to their containing whole components of the pathogen structure, these vaccines can elicit a vigorous immune response upon administration. However, there are some safety concerns, such as with live and inactivated vaccines, which may revert to pathogenic forms [2] or they can cause an allergic response [3]. Acellular/subunit vaccines are constructed from single components of the whole pathogen, which may be proteins [4], polysaccharides [5, 6], or pathogenic toxins [7] that may be found either on the surface of the pathogen cell wall/envelope, or they may be part of its internal structure. Unlike whole-cell vaccines, acellular vaccines do not have the same safety concerns, and they are more reproducible by recombinant DNA technology that simplifies manufacturing [8]. Although using a fragment of the whole structure is advantageous in terms of safety, it has the limitation of lower immunogenicity. More recently, nucleic acid-based vaccines (DNA or mRNA) encode immunogenic proteins of the pathogen structure. Upon vaccination with the DNA or mRNA, the host translates them into the immunogenic proteins, thus triggering an immune response. Although the nucleic acid-based vaccine approach is superior to cellular and acellular vaccines in terms of easy manufacturing and ability to quickly modify the vaccine in response to pathogen variant development, which we have seen for SARS CoV2, there can be concerns regarding low immunogenicity and lesser stability [9, 10]. The effect of antigen type and presentation, that is, protein vs DNA or mRNA, on the longevity of the immune response has yet to be well understood.

Among these vaccine groups, current subunit and nucleic acid-based vaccine strategies are more attractive approaches for developing novel vaccines. To this end, a great deal of attention is paid to overcoming the challenges that are faced by those approaches. For this purpose, taking advantage of the opportunities that modern pharmaceutical formulation and drug delivery technology provide is promising, such as particulate and non-particulate delivery systems to overcome their unique limitations. A variety of polymeric, lipidic, and inorganic delivery systems have been developed for vaccines over the last 2-3 decades. The purpose of these carriers is to package and protect the antigen, to facilitate uptake into cells (either into antigen-presenting cells or those that will produce the protein, such as muscle), and to provide adjuvant activity. Adjuvant function is particularly necessary for acellular and nucleic acid-based vaccines. Chitosan-based non-particulate delivery systems have been less studied but have certain unique properties and thus will be the focus of this review.

Chitosan is manufactured by the alkaline deacetylation of chitin, which is one of the most abundant polymers in nature. It is found in the shells of crustaceans such as shrimp, for example. As the result of alkaline deacetylation, the number of N-acetyl-D-glucosamine units in the chitin structure decreases and they are converted to β-1,4-D-glucosamine. Thus, chitosan is comprised of cationic β-1,4 linked monomers of D-glucosamine and N-acetyl-D-glucosamine [11, 12].

**FIGURE 1:**
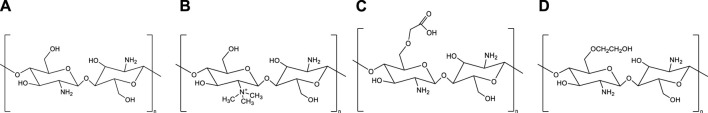
Chemical structures of chitosan and its derivatives. **(A)** Chitosan, **(B)** Trimethyl chitosan, **(C)** Carboxymethyl chitosan **(D)** Glycol chitosan.

Chitosan’s biological and physicochemical properties are dependent on its degree of deacetylation and endcapping. An increase in the deacetylation degree leads to an increase in the ratio of D-glucosamine units to N-acetyl-D-glucosamine units in the chitosan backbone. The D-glucosamine unit has an amine group (-NH_2_) that is protonizable, which gives chitosan some unique features, such as mucoadhesive properties via electrostatic interactions and enhanced cell permeability, making it more versatile compared to chitin. Furthermore, the water solubility of chitosan at slightly acidic pH is attributed to the protonation of the amine groups [13]. Amine groups also enable the chitosan backbone to be chemically modifiable; therefore, by adding different chemical groups to the chitosan structure, chitosan derivatives can be obtained that can increase the range of pharmaceutical properties and applications of chitosan. Besides amine groups, chitosan derivatives can also be synthesized by modifying the hydroxyl groups (-OH) of chitosan. Among the various chitosan derivatives, trimethyl chitosan (TMC), glycol chitosan, and carboxymethyl chitosan (Figure 1) are examples that have most frequently been studied for vaccine applications [14–16].

**FIGURE 2:**
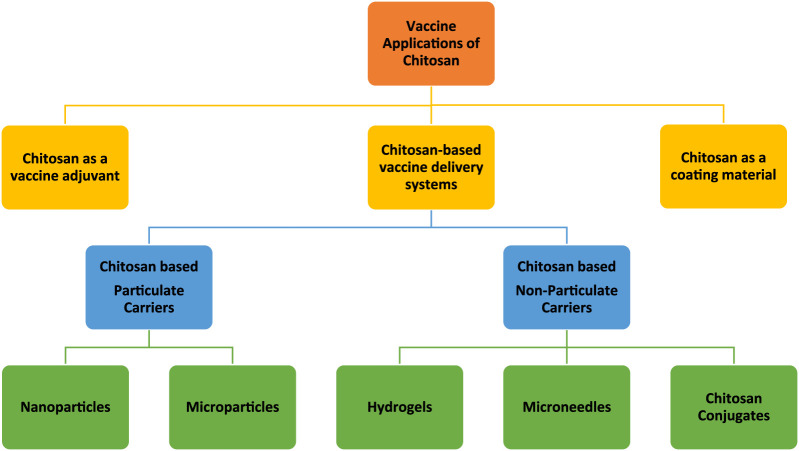
Schematic representation of vaccine applications of chitosan and its derivatives in different forms (in this figure the word “chitosan” stands for both chitosan and chitosan derivatives).

**FIGURE 3:**
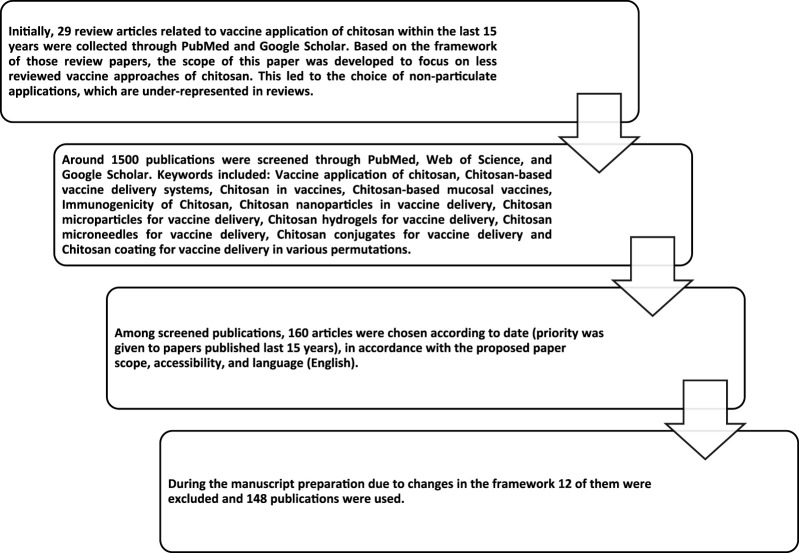
Flow chart of the reference collection process.

Although each derivative has its own unique features, in general, chitosan and/or chitosan derivatives are biodegradable, biocompatible, and non-toxic and have the potential for vaccine delivery applications in various forms (Figure 2). Chitosan-based particulate (chitosan nano- and microparticles) carriers have been the most extensively studied. Therefore, the vaccine application of those particulate delivery systems has been the focus of several comprehensive review papers [17–20]. In this review, chitosan-based particulate delivery systems will only be reviewed from a general perspective to highlight their promising properties that may help to overcome some of the challenges of today’s vaccine development. However, the focus of this review will be the usage of chitosan as: a) a vaccine adjuvant based on its intrinsic immunogenicity; b) non-particulate delivery systems; and c) a coating material. For this purpose, research papers published on these topics within the last 15 years were compiled as described in Figure 3.

## Immunomodulatory effect of chitosan and its adjuvant application

The rationale behind the usage of chitosan to facilitate vaccine delivery is not only because of its carrier properties but also because of its immune-triggering ability [21–24]. Several investigations have been carried out regarding its mode of action [25, 26]. According to a recent investigation, carried out by Carroll et al. (2016), chitosan induces mitochondrial stress that increases the permeability of the mitochondrial membrane [27]. As a result of this, mitochondrial factors such as mitochondrial DNA (mtDNA) diffuse into the cytosol. Released mtDNA molecules bind to an enzyme called cyclic-di-GMP-AMP synthase (cGAS). Upon binding to mtDNA, cGAS catalyzes cyclic-di-GMP-AMP (cGAMP) synthesis from ATP and GTP. Then, the newly synthesized cGAMP binds to an adaptor protein, a stimulator of IFN genes (STING), which leads to the activation of STING and, subsequently production of type I IFN [27]. Type I IFNs play a role in the generation of important immune responses. For instance, they stimulate a Th1-biased CD4^+^ cell response and promote activation, maturation, and migration of dendritic cells (DCs). Thus, chitosan induces a Th1-biased immune response [26, 27], which is essential for an anti-viral immune response for example.

Chitosan not only induces cellular immunity but also humoral immunity [27–29]. Application of a chitosan solution together with a model protein antigen, β-galactosidase, significantly increased antibody titers and CD4^+^ proliferation, demonstrating that chitosan can elicit both cellular and humoral immune responses in a mouse model [28]. Because of this potential for immunomodulatory activities, chitosan’s usage as a vaccine adjuvant has been explored. For instance, the application of chitosan glutamate as an adjuvant for inactivated poliovirus vaccines significantly increased the immune response to poliovirus in comparison to non-adjuvanted vaccines upon intramuscular administration to mice [30]. In another study, chitosan was tested as an adjuvant for H5N1 subunit protein, hemagglutinin, for intranasal influenza vaccines. Vaccination of mice with chitosan adjuvanted vaccines increased production of TNF-α, IFN-γ, and IL-2 cytokines which demonstrates Th1 cell-based immune response as well as production of IL-4, IL-5, and IL-10 cytokines that are associated with Th2 based immune response. Thus, using chitosan as an adjuvant elicited a mixed Th1/Th2 type CD4^+^ T cell immune response that was superior to the non-adjuvanted vaccine formulations [31]. Eliciting a mixed immune response of Th1 and Th2 polarization is desirable; while Th1 cytokines are associated with the generation of the cellular immune response, Th2 cytokines take a role in the generation of humoral (antibody) immune response [32]. Mann *et al.*, (2014) tested chitosan derivatives (glutamate and TMC) as vaccine adjuvants for hemagglutinin subunit protein to develop intranasal/intratracheal influenza vaccines for highly pathogenic avian influenza [33]. Vaccine formulations consisted of simple aqueous mixing of either glutamate chitosan or TMC with hemagglutinin. According to their results, these chitosan derivatives increased the protective immune response and fully protected ferrets, which model the clinical aspects of human influenza infection, against a lethal challenge with live virus infection [33]. Chitosan can also enhance the effect of other adjuvants when used in combination. For instance, a combination of chitosan with an adjuvant, cytosine-phosphate-guanine oligodeoxynucleotide (CpG), elicited higher Th1 and Th17 cell responses compared to CpG alone. Likewise, a chitosan-CpG combination was even more efficient than the combination of CpG with a conventional alum adjuvant in terms of promoting both Th1 and Th17 immune responses [34]. Th17 immune response eliciting feature of chitosan has also been observed in other studies [31, 35–37]. Th17-mediated response is essential for protecting against infections. It leads to the recruitment of neutrophils, which are responsible for eliminating pathogens, and stimulates the secretion of anti-microbial peptides by mucosal epithelial cells to fight against harmful microorganisms [37–39]. However, at this point, it is essential to note that some safety issues may arise due to the activation of Th17-mediated immune responses. Th17 cells produce specific cytokines, including IL-17, IL-17F, IL-21, and IL-22. Reports suggest that the pro-inflammatory cytokines IL-17 and IL-22 can lead to inflammation, which poses a safety concern for vaccine components that trigger the Th17 response. Therefore, further investigation is required to determine if the Th17-eliciting mechanism of chitosan can cause inflammation [38, 39].

These abovementioned studies demonstrate that chitosan and its derivatives are promising adjuvant and co-adjuvant candidates for diverse vaccine applications, including subunit vaccines. Co-adjuvant designs should include a thorough understanding of the mechanism of action of each adjuvant to produce a vaccine with complementary and appropriate immune activation. For example, a Th1 response is desirable for vaccines against viral infections so as to generate a cellular response against virally infected cells upon exposure to the viral pathogen. This may not be as essential for extracellular pathogens such as bacteria, however, a strong antibody response would be needed. Other organisms have complex lifecycles, thus requiring both. In general, however, a balanced cellular and humoral response can be most effective.

## Chitosan-based particulate carriers for vaccines

### Expectations from novel vaccine carriers

**FIGURE 4:**
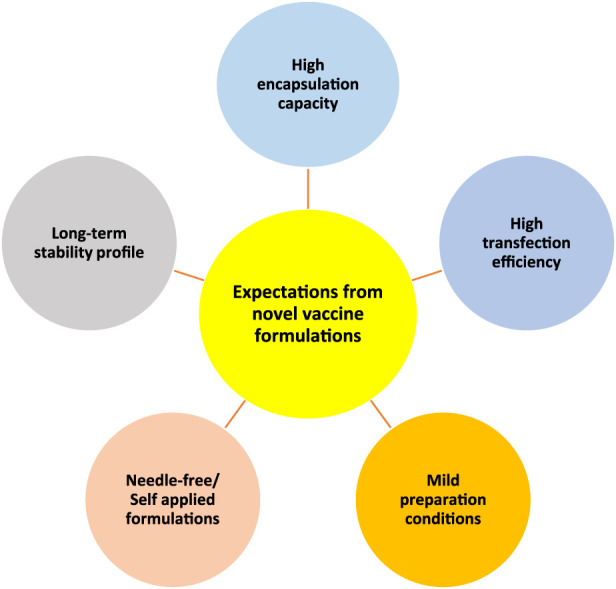
Schematic representation of five main expectations from novel vaccine formulations/delivery systems. High encapsulation capacity, high transfection efficiency, mild preparation conditions, needle-free/self-applied formulations, and long-term stability profile.

Generally, by considering different vaccine types, we have listed 5 major expectations that need to be satisfied by novel vaccine formulations (Figure 4). Each vaccine type has some limitations that need to be overcome by different strategies to satisfy those expectations. For instance, for subunit vaccines, the delivery of adjuvants along with antigens in the particulate delivery system is crucial [40], while for nucleic acid-based vaccines efficient transfection into cells is vital [41]. To this end, particulate carriers need to have a high loading capacity to be able to carry subunit vaccine payloads whereas having efficient interaction and penetration ability with cell membranes to increase the transfection of genetic materials into cells is vital for nucleic acid-based vaccine delivery systems. Furthermore, the usage of harsh conditions or organic solvents can be damaging for vaccine payloads therefore mild preparation conditions are also necessary [42]. Additionally, making vaccines more patient-compliant and accessible for everybody is also another expectation from novel vaccine formulations. To achieve this, it is necessary to develop needle-free vaccine formulations that can be self-administered, such as nasal sprays [43] and microneedles. On the other hand, many of the currently available vaccines require cold chain delivery, which is often not feasible or affordable for many countries therefore their stability needs to be improved [44–46]. All in all, there is a need for improved vaccine delivery systems that can meet these requirements.

### Effectiveness of chitosan particulate carriers to overcome the challenges of vaccine delivery

The high encapsulation capacity of chitosan-based particulates for subunit vaccines has been demonstrated in several studies. Bal *et al.*, (2012) tested TMC as a vaccine delivery system using ovalbumin (OVA) as a test antigen along with different adjuvants such as lipopolysaccharide (LPS), PAM3CSK4 (PAM), CpG DNA, muramyl dipeptide (MDP) and the cholera toxin B subunit (CTB). This study showed that although each of those adjuvants has a different chemical nature, chitosan nanoparticles can encapsulate each of them and the antigen together with high efficiency [47]. Furthermore, Xu *et al.*, (2022) were able to encapsulate three different malaria antigens (Plasmodium falciparum malaria parasite, apical membrane antigen (PfAMA- 1), merozoite surface antigen (PfMSP-1), pre-erythrocytic stage antigen (PfCSP)) together in layer-by-layer TMC nanoparticles with total antigen encapsulation efficiency >90% [16]. Furthermore, they demonstrated that the chitosan encapsulation protected antigen integrity and maintained the immune triggering ability of each antigen when they tested *in vivo* on mice models [16]. These are just a few examples of studies that illustrate the high and versatile vaccine payload loading capacity of chitosan nanoparticles.

Chitosan-based nanoparticles have also been as extensively evaluated as nucleic acid-based vaccine carriers to overcome the challenges of those vaccines. Due to its cationic nature, chitosan-based nanoparticles electrostatically interact with negatively charged cell membranes, and this increases cellular uptake of nanoparticles and thus improves the transfection of nucleic acid-based vaccines [48–50].

**FIGURE 5:**
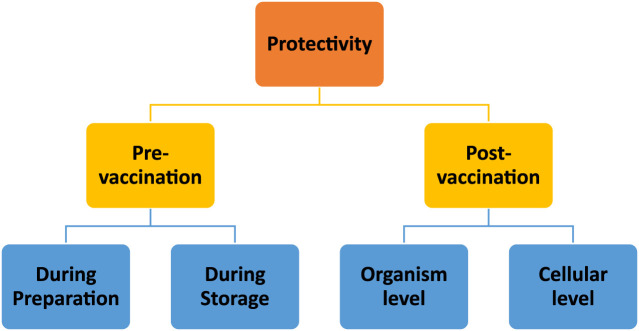
Schematic representation of evaluation of chitosan nanoparticles’ protective abilities at the stages of pre- and post-vaccination periods.

Due to their high protective effect on their payloads, chitosan-based delivery systems are also accepted as safe carriers for both subunit and nucleic acid vaccine delivery. This protective profile can be evaluated in two periods (Figure 5): pre- and post-vaccination.

The pre-vaccination period can also be considered in two stages: product preparation and storage. Firstly, chitosan nanoparticle fabrication methods [51] and production must be designed with care to preserve the integrity of the antigen(s) or nucleic acid. Fortunately, chitosan nanoparticle preparation methods do not typically involve harsh conditions [52]. In terms of stability during the storage period, promising results were obtained in several research studies. For instance, Prego *et al.* (2010) reported that recombinant hepatitis B surface (rHBs) antigen encapsulated in chitosan nanoparticles can preserve the integrity of the rHBs antigen for up to 3 months [53]. Cordeiro *et al.* (2021) tested the thermostability of chitosan nanoparticles in an accelerated stability test conducted at 40°C and 75% humidity. According to their results, freeze-dried chitosan nanoparticles protected the rHBs antigen for 1 month under those conditions [54]. As well, chitosan nanoparticles exhibited a protective effect when used as a carrier for a peptide-based antigen along with a nucleic-acid-based adjuvant, polyinosinic-polycytidylic acid [poly (I:C)] [55].

The post-vaccination period also involves protectivity at two levels: protection at the cellular level and at the organism level. Protection of the antigen at the cellular level is crucial to avoid premature degradation. The protective effect of chitosan nanoparticles is mainly attributed to the presence of amine groups on the chitosan backbone that have H^+^-buffering capacity. H^+^ buffering is crucial, especially during cellular uptake, because it leads to osmotic swelling of endosomes during the internalization of nanoparticles, including by the target antigen-presenting cells. The swelling of endosomes leads to disruption of the endosomal membranes, and particles can then escape to the prenuclear area, thus protecting the payload from the acidic medium of the endosomes [52, 56–58]. Chitosan nanoparticles afford protection to their vaccine payloads at the organism level, as well, for example, considering intranasal or oral administration. It has been demonstrated that chitosan nanoparticles can protect vaccines not only in the intranasal environment but also in the harsh gastrointestinal tract. In this regard, Le *et al.* (2009), reported that chitosan nanoparticles can protect vaccine payloads from the degrading effects of gastrointestinal conditions upon oral administration and lead to an enhanced immune response in a mouse model [59]. A similar protective effect against low pH and enzymatic degradation was also obtained with chitosan microparticles when they were used for oral DNA vaccine delivery [60].

On the other hand, due to their mucoadhesive and penetration enhancement properties, chitosan-based delivery systems hold great promise for the development of needle-free vaccines (e.g., intranasal vaccines). The mucoadhesive nature of chitosan is attributed to its ability to electrostatically interact with the negatively charged glycoproteins and polysaccharides found in mucus. This binding to mucus prolongs the retention time of nanoparticles on the mucosal surface and prolongs the time needed to encounter antigen-presenting cells scavenging the mucosal surface [61]. A significant barrier, however, is physiological nasal clearance mechanisms. Due to this phenomenon, naked vaccines cannot penetrate in sufficient quantity into the nasal-associated lymphoid tissue (NALT), where immune cells can be found. Thanks to its high penetration enhancement capacity, chitosan enhances the delivery of antigens across mucosal barriers into the NALT [62].

All in all, chitosan-based delivery systems satisfy the five expectations of an effective vaccine delivery system as demonstrated in Figure 4. Therefore, chitosan and its derivatives should be considered in vaccine development programs, especially for challenging routes of administration, such as intranasal and oral.

## Chitosan-based non-particulate vaccine delivery systems

Three non-particulate vaccine delivery systems based on chitosan will be considered here, as they represent the bulk of current investigations. These include chitosan in hydrogels, microneedles, and molecular conjugates.

### Chitosan hydrogels

**FIGURE 6:**
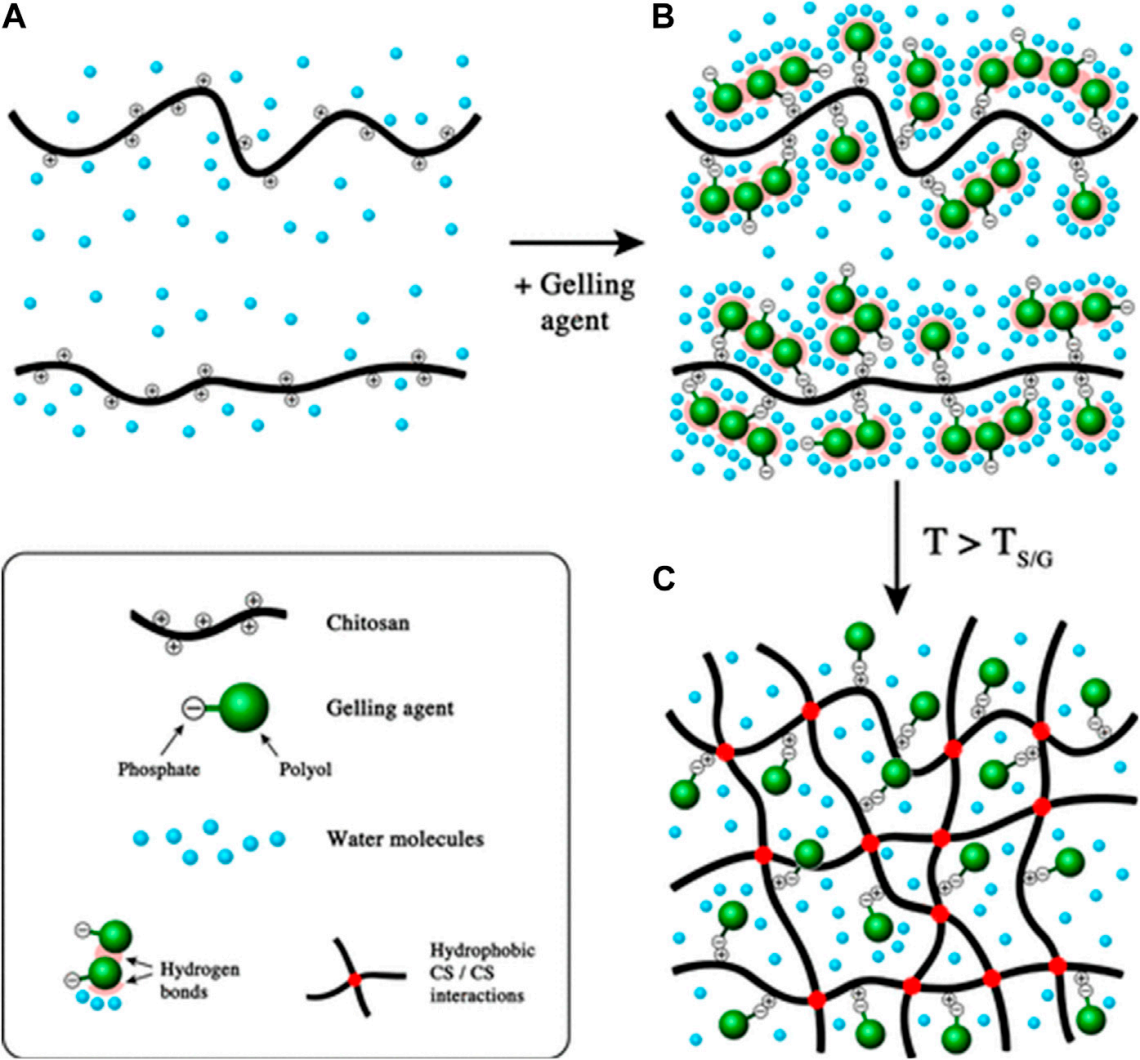
Gel formation mechanism of thermosensitive chitosan hydrogels as the function of increased temperature. **(A)** chitosan solution; **(B)** mixture of chitosan solution and gelling agent at ambient temperature; **(C)** formation of chitosan hydrogels above T_S/G_ (sol/gel transition temperature). Adapted with permission from Supper S, Anton N, Seidel N, Riemenschnitter M, Schoch C, Vandamme T. Rheological study of chitosan/polyol-phosphate systems: Influence of the polyol part on the thermo-induced gelation mechanism. Langmuir. 2013; 29 (32): 10229–37. DOI: 10.1021/la401993q. Copyright 2013 American Chemical Society.

Hydrogels are dosage forms that contain a high-water content that is formed by the crosslinking of highly hydrophilic polymers. Various crosslinkers (gelling agents) are available to modulate the release properties of the hydrogels [63]. One of the extensively studied types of hydrogels is thermosensitive hydrogels, which are liquid at room temperature. However, upon injection into the body, the increased temperature causes a transition of the hydrogel from liquid to solid. That transition is attributed to a decrease in the electrostatic interactions between the polymer and its crosslinker and the formation of hydrophobic interaction between the polymer chains upon dehydration, which occurs as a function of increasing temperature [63, 64] (Figure 6). Over time, the semi-solid gel structure is exposed to degradative enzymes (e.g., lysozyme) and breaks down. As they lose their structural integrity, the entrapped payload gradually leaks into the administration site, which creates a sustained release depot. That type of sustained release is thought to be important for mucosal vaccines, in theory, by enhancing the opportunity for dendritic cell sampling [65].

Due to their biodegradable and biocompatible nature, chitosan-based hydrogels are attractive candidates for vaccine delivery applications. Furthermore, their payload encapsulation capacities not only allow them to incorporate antigen/adjuvant but also particulate carriers. Because of these versatile properties, chitosan hydrogels are being explored for both human [66–68] and veterinary vaccines [69–72].

#### Chitosan hydrogels as antigen and adjuvant carrier

Gordon *et al.* (2008) conducted a comparative study between antigen-loaded chitosan nanoparticles vs. chitosan hydrogels regarding their release and immunostimulatory profiles [73]. In that study, ovalbumin was used as the model antigen. As expected, OVA was released from chitosan nanoparticles faster than the chitosan hydrogel, such that over 10 days, they noted that 50% of the encapsulated antigen was released from the nanoparticles compared to only 10% with the hydrogel. Interestingly, while both strong humoral and cellular immune responses were obtained with the chitosan hydrogel (comparable to that obtained with their positive control, alum +OVA), no significant immune enhancement effect was observed with OVA-loaded chitosan nanoparticles. This was attributed to the administration route, which was subcutaneous, where the hydrogel could achieve a depot effect, whereas nanoparticles lacked this ability [73]. Similarly, strong cellular and humoral immune responses were also observed in another comparative study in which chitosan hydrogels were loaded with OVA along with an adjuvant [Granulocyte-macrophage colony-stimulating factor (GM-CSF)]. In that study, a chitosan/OVA/GM-CSF hydrogel formulation was compared with the commercially available complete Freund’s adjuvant or incomplete Freund’s adjuvant. The chitosan hydrogel loaded with OVA/GM-CSF elicited significantly higher OVA-specific IgG expression as well as OVA-specific CD4^+^ and CD8+T immune responses than these commercial adjuvants upon subcutaneous administration [74].

Wei *et al.* (2017) developed a pH-sensitive hydrogel based on phosphorylated chitosan (PCS) that forms a hydrogel at pH > 7.0. and incorporated OVA as the model antigen [75]. Hydrogel formulations were administered intramuscularly, and the release of the antigen at the immunization site was visualized by fluorescent microscopy. Imaging demonstrated that a controlled release of the antigen was occurring at the injection site over 10 days, and approximately 67% of the antigen was released during that period in a controlled manner, whereas after 5 days, no antigen was observed in the injection site when OVA was administered alone as the negative control. Regarding the immune response outcome, PCS hydrogels increased serum IgG antibody levels and secretion of cytokines IFN-γ and IL-4, and they also triggered robust CD4^+^ T and CD8^+^ T cell responses [75].

Wu *et al.* (2012) developed a chitosan derivative, N-[(2-hydroxy-3-trimethylammonium) propyl] chitosan chloride (HTCC), for use in a thermosensitive hydrogel for intranasal vaccine delivery [76]. One of the aims of the study was to develop an alternative delivery system for particulate vaccines with short mucosal retention times. Zaire *Ebola* virus antigen was encapsulated in the hydrogel, which was able to retain 31% of the antigen payload in the nasal cavity over 3 h compared to 8% for the naked antigen. In terms of the immune response outcome, upon intranasal administration of the hydrogel to mice, secretion of mucosal secretory IgA (sIgA) antibody was increased compared to non-hydrogel formulated antigen. Improving sIgA is essential to protect host mucosal surfaces from infection by pathogen binding and subsequent immune clearance. Additionally, the secretions of type I cytokines (IFN-c and IL-2) were upregulated, resulting in a Th1-polarized immune response. The titers of IgG1 and IgG2 antibodies were 3-fold higher with the hydrogel vaccines in comparison to that of the control antigen [76]. Similarly, in a second study that the same research group conducted, H5N1 split antigen in this same type of hydrogel delivery system improved the immune response compared to non-formulated antigen [77]. They also evaluated hydrogels prepared with HTCC of different quaternization degrees (QDs; 0%, 21%, 41%, 60%, and 80%) to determine the effect of the hydrogel structure on its immunogenicity [78]. QD has a direct positive impact on the positive charge and hydrophilicity of the chitosan chain, which in turn influences the physicochemical properties of hydrogels, such as gelation time, retention time, antigen entrapment, and viscosity. According to the research results, hydrogels with an average QD (41%) had optimal local and systemic immune response outcomes. The observed phenomenon was attributed to the collective influence of positive charge, viscosity, and gelation time at the QD of 41%. Since the positive charges of hydrogels with QDs (0% and 21%) are lower than that of hydrogels with QD 41%, their binding affinity to antigen and mucus is lower, which decreases penetration of the antigen into NALT. On the other hand, hydrogels with 41% QD had a gelation time of 21 ± 2 min, while hydrogels with higher QD (60%, 80%) had a longer gelation time of above 1 h. Due to low viscosity and long gelation time, nasal retention of hydrogels (with QD of 60%, 80%) decreases, resulting in lower antigen delivery into the NALT [78].

In another study, Bedford *et al.* (2020) developed an influenza antigen (nucleoprotein peptide) and LPS adjuvant-loaded chitosan hydrogel formulation and tested it intranasally as a booster dose for mice that received a priming dose intraperitoneally [79]. It was reported that the number of tissue-resident memory CD8^+^ T increased 10-fold as a result of hydrogel vaccine administration. Furthermore, upon challenge with live influenza virus, it was observed that the mice which received the intranasal hydrogel booster dose had a 100-fold lower pathogen load in the nasal cavity. Notably, the lungs of 80% of mice receiving the hydrogel intranasal booster were completely protected from the viral infection that causes pneumonia [79].These studies demonstrate that chitosan-based hydrogels can be efficiently designed with different properties to deliver various antigen and adjuvant payloads.

#### Chitosan hydrogels as particulate vaccine carriers

Chitosan-based hydrogels have also been used in combination with polymeric nanoparticles for additional functionality in vaccine applications. For instance, Bobbala *et al.* (2018) incorporated antigen-loaded poly (lactic-co-glycolic acid) (PLGA) nanoparticles in chitosan hydrogels [80], while Gordon *et al.* (2012) used chitosan hydrogels for liposome and cubosome-based particulate vaccines [81]. Recently, chitosan hydrogels have been applied to deliver plant-based nanoparticulate vaccines [82, 83]. For instance, chitosan hydrogels were loaded with plant-derived cowpea mosaic virus (CPMV) nanoparticles that were conjugated to coronavirus 2 spike protein epitope 826 by Nkanga and coworkers [83]. They tested this system in mice and demonstrated that incorporation of 826-CPMV conjugate nanoparticles in chitosan hydrogels resulted in a prolonged immune response (20 weeks), and it generated a shift in the type of immune response from Th1 to Th2, whereas with 826-CPMV conjugate nanoparticles alone, a Th1-biased immune response was observed; this difference was attributed to the increased residence time that alters interaction of particles with antigen-presenting cells. Such that upon administration, particles easily transit to lymph nodes and trigger B-cells that lead to Th1-biased immune response generation. At the same time, this process was followed by the interaction of particles that were released later (from hydrogel) with antigen-presenting cells that resulted in eliciting a Th2 immune response over time [83]. As mentioned above, this type of release pattern may be optimized for a self-boosting vaccine delivery system; clearly, the release rate and extent are related to the type of response generated.

Although the abovementioned studies demonstrate promising aspects of chitosan-based hydrogels for both antigen and particulate system carrying, it is noteworthy that chitosan-based thermosensitive hydrogels have not yet been extensively studied for vaccine application. Therefore, further comprehensive studies should be carried out to investigate the limitations of thermosensitive hydrogels. For example, a weak temperature response may result in delayed gelation and weak mechanical properties, which in turn could dramatically shift the immune response due to reduced antigen retention. The gelation agent and ionic content, such as salt concentration, must be compatible with local tissues at the site of administration, which restricts the choices of reaction components. Burst release is a commonly encountered challenge for any polymeric delivery [84–86], which, in the case of vaccines may represent wasted antigens. Application of this technology to vaccines is a relatively recent advance, thus further studies are needed to explore how the loading and release properties of thermosensitive chitosan hydrogels can be optimized to enhance and direct the immune response in the context of specific antigens, such as specific pathogen-associated proteins or whole inactivated pathogens.

### Chitosan microneedles

**FIGURE 7:**
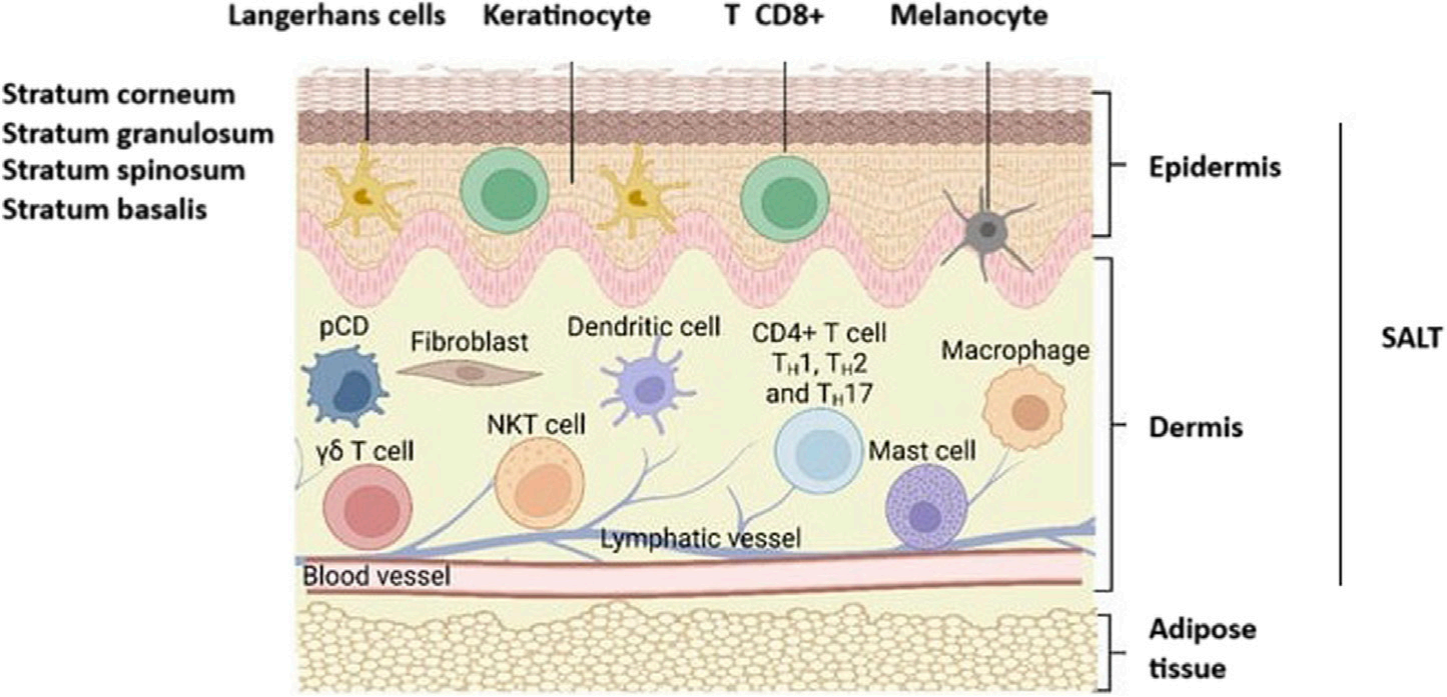
Representation of skin anatomy and the immune cells found in SALT (Skin-Associated Lymphoid Tissues). CD8^+^ T, CD8^+^ cytotoxic T cells; CD4^+^ TH1, TH2, and TH17, T-γδ subsets of T cells; pDC, plasmacytoid dendritic cells; NKT, Natural killer T cells. Adopted from [89] and slightly modified.

Due to the immune cell-rich nature of Skin-Associated Lymphoid Tissues (SALT), vaccination in this region is attractive. The SALT region covers the dermis and the epidermis layer [87]. The epidermis consists of different layers and the outermost layer is the stratum corneum [88] (Figure 7). Thus, to reach the SALT region, passing through the stratum corneum, which has a thickness of 10–20 μm, is needed [89]. “Microneedles” are micron-sized drug delivery vehicles that can penetrate through the stratum corneum and release their payload under this layer (into the epidermis and/or the dermis) without causing pain. Therefore, they are attractive carriers for vaccine delivery. As vaccine carriers, microneedles have several advantages, such as the potential to release their payload in a controlled manner and by creating a depot effect that is important for vaccine efficacy. Furthermore, microneedles may improve the stability of vaccine formulations and eliminate both the need for cold storage and parenteral administration and its cost-intensive supplies [90–92]. Microneedles may even improve patient acceptability for needle-phobic patients and improve access in locations lacking adequate medical personnel for performing injections.

**FIGURE 8:**
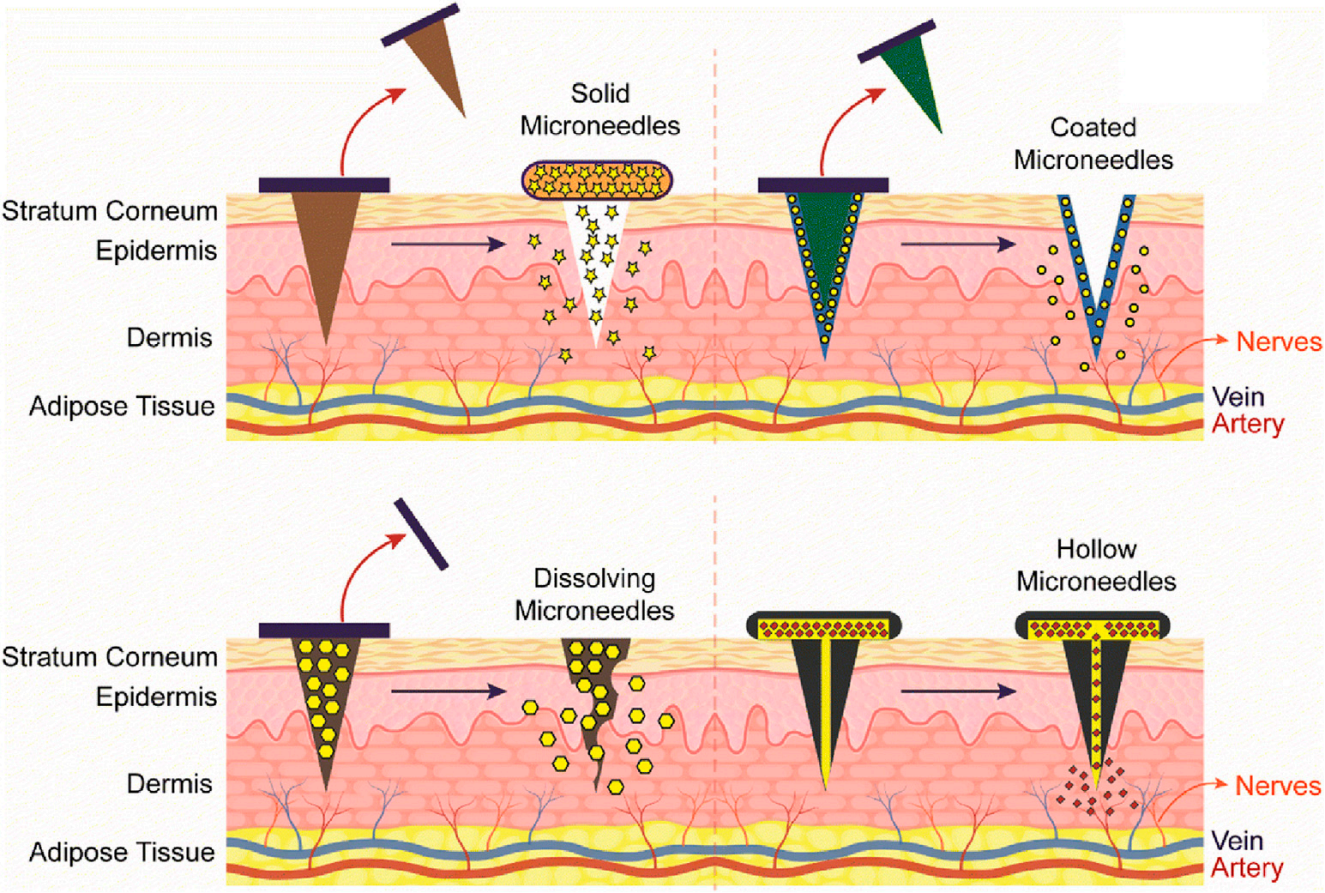
Representation of different type of Chitosan microneedles (solid, coated, dissolving, and hollow), their administration into the skin and release mechanisms upon administration. Adopted from [95].

Several microneedle types are available to allow flexibility in the delivery of various antigens. Solid, hollow, dissolving, and coated microneedle types are examples that are frequently used for this purpose [93–95] (Figure 8). Among them, chitosan-based dissolving and coated microneedle types have been developed to deliver vaccine antigens.

#### Dissolvable chitosan microneedles

To prepare dissolvable microneedles, typically, the chitosan gel is poured into microneedle molds to form the main microneedle structure. Chen *et al.* (2013) developed an embeddable chitosan microneedle system that incorporated OVA as a model antigen and tested its immunogenicity in rats [96]. They reported that the holes that formed in the skin upon microneedle insertion resealed quickly in about 1 h. In terms of the antigen’s *in vivo* release profile, the degradation of the chitosan microneedles occurred over approximately 2 weeks, with a gradual release of the antigen. Regarding the immune response outcome, it was observed that vaccination with chitosan microneedles triggered a significantly higher immune response compared to intramuscular immunization with the same antigen (unadjuvanted). A robust immune response was evoked with the chitosan microneedles which lasted for 6 weeks [96]. In their second study, this research group developed a dissolvable patch containing chitosan microneedles loaded with OVA [97]. Upon application of the microneedle patch, holes in the skin closed completely after 6 hours, and a small amount of irritation was observed that disappeared after 1 day. These differences compared to their previous formulation may be attributed to the dissolvable patch, considering that the applicator patch was detachable in the original formulation. Degradation of the microneedles occurred over approximately 4 weeks, illustrating that the new design maintained a depot effect. Incorporation of OVA antigen in the chitosan microneedles elicited a significantly higher immune response in comparison to intramuscularly administered antigen; the antigen-specific immune response: OVA-specific IgG antibody levels persisted for 18 weeks. An additional benefit was observed in that when a 2.5-fold lower antigen amount (200 μg) was used, the microneedles elicited a higher immune response than intramuscularly administered full-dose (500 μg) OVA [97]. Achieving an antigen-sparing effect is one of the goals of vaccine delivery in order to reduce the vaccine cost. In their subsequent study, the microneedle formulation was loaded with influenza hemagglutination protein, and it was observed that vaccination with the chitosan microneedles protected all animals from live influenza virus challenge (0% mortality): mortality rates were 60% and 100% in the intramuscularly vaccinated mice group and non-immunized mice group, respectively [98]. The same research group also showed that chitosan microneedles can be designed in combination with other polymers, such as hyaluronic acid, to achieve a biphasic release of the antigen from the microneedles [99]. The biphasic release profile is advantageous because it starts with a fast-release process that induces an enhanced primary immune response followed by a sustained release process that increases the exposure time of the antigens to the antigen-presenting cells. According to the result of this study, antigen-loaded chitosan-hyaluronic microneedles had a significantly higher immunogenicity than antigen-loaded chitosan-only microneedles. It was also revealed that vaccination with a single dose of chitosan-hyaluronic microneedles (contains 100 μg OVA) had a considerably better immune response outcome than two doses (2 × 100 μg) of non-formulated OVA antigen that were administered subcutaneously as prime and booster doses. These results imply a dual role for chitosan as an adjuvant and a controller of the antigen release rate. Thus, this study showed that chitosan-based microneedles are also promising to develop potentially single-dose vaccine formulations that would be practically and economically advantageous [99]. Note that the release rate would require tuning to the specific antigen due to the anticipated variation in the timing of the primary and secondary responses to a particular antigen.

Processing of both antigen and adjuvant in the same antigen-presenting cell (APC) is necessary for the primary immune response, therefore, both should be co-delivered to the APC [100, 101]. To achieve this with microneedles, antigen/adjuvant-loaded nanoparticles can be incorporated into chitosan microneedles. Li *et al.* (2020) encapsulated OVA antigen and CpG adjuvant in chitosan nanoparticles and embedded the nanoparticles into microneedles comprised of polyvinylpyrrolidone (PVP) [102]. One of the rationales given for nanoparticle encapsulation in microneedles was to deliver antigen/adjuvant to regional lymph nodes to enhance the immune response outcome. Following local administration, particles released from the microneedles migrated to and accumulated in nearby lymph nodes. This efficient lymph node trafficking was attributed to the small size (60 nm) of the nanoparticles, which is optimal for migration through tissue lymphatic transport pathways. The positive surface charge of nanoparticles subsequently enables the uptake and activation of the APCs. This lymph node-specific antigen/adjuvant delivery promoted a robust immune response that had a similar magnitude as compared to subcutaneously injected nanoparticle formulation [102] but administered in a needle-free dosage form. In another study, adjuvant and mannose-modified chitosan nanoparticles (loaded with OVA) were encapsulated in chitosan microneedles to actively target dendritic cells, which have mannose receptors on their membranes for facilitating internalization of the particles. As a result, increased uptake of nanoparticles by dendritic cells was observed *in vitro* as well as an increased maturation rate of dendritic cells when they were tested *in vivo* [103].

#### Coated chitosan microneedles

For coated microneedles, chitosan is added to preformed microneedle arrays in a layer-by-layer format, entrapping the antigen between those layers. Van Der Maaden et al. (2015) combined inactivated polio vaccine (IPV) antigen with TMC [104] in a coated microneedle dosage form. Due to the opposing charges of IPV antigen and TMC, binding occurs electrostatically, and layers can be deposited one on top of the other. This approach would require an antigen with significant regions that are anionic in nature, which may be a limitation. *In vivo*, immunization with this microneedle formulation elicited IPV-specific immune responses in rats [104]. In another study, diphtheria toxoid (DT) was loaded in coated microneedles [105]. It was revealed that the DT loading can be increased by increasing the number of layers. The ability to fine-tune the antigen dose is necessary for optimizing the immune response to the desired magnitude. Upon intradermal immunization of mice with microneedles that carried 0.6 µg DT, the DT-specific IgG titers were similar to the control group, which were subcutaneously immunized with 5 µg of DT with alum adjuvant. This showed that TMC-coated microneedles are efficient delivery vehicles for antigens to elicit DT-specific immune responses with low antigen doses [105]. TMC layers have been used not only for the entrapment of free antigens but also for antigen-loaded nanoparticles within the microneedle structure as described above. For instance, Du *et al.* (2018) successfully used the same method to entrap lipid bilayer-coated mesoporous silica nanoparticles loaded with DT (LB-MSN-DT) in between the TMC layers of the microneedles [106].

Table 1 provides recent examples of published research studies demonstrating that chitosan microneedles are promising candidates for vaccine delivery because of their vaccine delivery potential, including examples of employing adjuvants or nanoparticle strategies. The combination of chitosan microneedles with nanoparticles enables control over the antigen release rate, targets tissues and APCs, and facilitates co-delivery of antigen and adjuvant, all of which are critical factors in being able to modulate the subsequent immune response upon vaccination. Thus, the microneedle-nanoparticle combination should be designed carefully, paying particular attention to the timing of antigen release. The type of microneedle is also a key factor. For instance, intriguingly, neither the combination of antigen-loaded chitosan nanoparticles with dissolvable chitosan microneedles [103] nor the combination of mesoporous silica nanoparticles with coated chitosan microneedles [106] improved their overall immunogenicity in comparison to naked antigens delivered by chitosan microneedles [103, 106]. However, the incorporation of mesoporous silica nanoparticles into hollow microneedles instead of coated microneedles increased the immune response [106]. These results demonstrate that the type of the microneedle itself should be considered during the development of nanoparticle-incorporated chitosan microneedle vaccines.

**TABLE 1:** Properties of chitosan-based microneedles and animal models used for *in vivo* immunization.

Microneedle Name	Microneedle composition	Supporting array	Payload	Length (μm)	Tested animal model	References
Fully embeddable microneedle	Chitosan	PLA	OVA	600	Rats	[96]
Patch-dissolvable microneedle	Chitosan	PVA/PVP	OVA	600	Rats	[97]
Implantable/patch-free microneedle	Chitosan	PVA/PVP	OVA/Hemagglutination unit	602 ± 22	Mice	[98]
Composite microneedle	Chitosan and Hyaluronic acid	PVA/PVP	OVA	550	Rats	[99]
Dissolving microneedle array	CS–OVA–CpG NPs and PVP	PVP	CS–OVA–CpG NPs	600	Mice	[102]
Dissolving microneedle patch	CS, CS–OVA NPs, and PVP	HPMC	CS–OVA NPs and *Bacillus* Calmette–Guerin polysaccharide	463 ± 8.29	Mice	[103]
pH-sensitive/Coated microneedle	TMC	Silicon	IPV	200	Rats	[104]
pH-sensitive/Coated microneedle	TMC	Silicon	DT	220	Mice	[105]
Coated microneedle	TMC	Silicon	LB-MSN-DT	200	Mice	[106]
Biodegradable microneedle patch	Chitosan and MSN-CpG/OVA-HBc	Chitosan	MSN-CpG/OVA-HBc	250	Mice	[107]

Abbreviations: PLA, poly(L-lactide-co-D,L-lactide); OVA, ovalbumin; PVA, polyvinyl alcohol; PVP, polyvinyl pyrrolidone; CS, chitosan; CpG ODN, cytosine-phosphate-guanine oligodeoxynucleotide; NPs, nanoparticles; TMC, trimethyl chitosan; HPMC, hydroxypropyl methylcellulose; IPV, inactivated polio vaccine; DT, diphtheria toxoid; LB, lipid bilayer; MSN, mesoporous silica nanoparticles; HBc, Hepatitis B core protein.

### Chitosan conjugates

Chitosan is also used as a conjugate for efficient delivery of subunit vaccines. Chitosan conjugate vaccines are formed by covalent binding of chitosan to the antigens directly. These structures have several advantages over both a simple mixture of adjuvant-antigen systems and antigen-loaded particulate systems. Those advantages include increasing the co-internalization of the antigen and adjuvant combination, minimizing side effects, improving mucosal penetration, and preventing antigens from aggregation [108, 109]. Example studies for some of those advantages are detailed in the following paragraphs of this section.

As mentioned above, codelivery of the antigen and adjuvant is necessary for the immune response, therefore they should be in close proximity to each other at the site of APC uptake, and this is inefficient or difficult to achieve for those vaccines consisting of a simple mixture of soluble antigen and adjuvant [100, 101]. On the other hand, high doses of vaccines may be needed to get a sufficient immune response with those formulations which do not provide for co-delivery of antigen and adjuvant, unlike conjugate vaccines. Due to its adjuvating property, the conjugation of chitosan to antigens eliminates the need for another adjuvant and increases the likelihood of co-internalization. Slütter *et al.* (2010) reported that the conjugation of OVA to TMC (OVA-TMC) increased its uptake by DCs significantly in comparison to OVA alone and a simple mixture of OVA and TMC (OVA/TMC) [110]. It was also reported that this increased uptake is an active process mediated by C-type lectin receptors rather than a passive diffusion. TMC-OVA was also superior to OVA/TMC and OVA-loaded TMC nanoparticle vaccines in terms of inducing dendritic cell maturation, as indicated by a greater expression of the CD86 cell surface marker. Meanwhile, OVA-TMC induced the highest IgG production, indicating the most potent immune response compared to other treatments (OVA, OVA/TMC, and TMC nanoparticles) [110]. Similarly, mucosal and humoral immune responses were obtained when chitotriose, a chitosan hydrolysate, was conjugated with a tumor-associated carbohydrate-based antigen [111]. Yu *et al.* (2016) tested a chitosan conjugate vaccine for protection against *Mycobacterium tuberculosis*. In that study, inulin was used along with chitosan as an adjuvant, and CFP_10_-TB_10.4_ fusion protein (CT) of tuberculosis was incorporated as the antigen [112]. It was revealed that the chitosan-inulin conjugate had a synergistic adjuvant effect that triggered a significantly greater immune response in terms of Th1-type cytokines (IFN-γ, TNF-α, and IL-2) and Th2-type cytokine (IL-4) production as well as CT-specific IgG titer when conjugated to CT in comparison to the chitosan-CT or inulin-CT conjugates [112]. This study demonstrated that chitosan conjugate vaccines can also be designed in a way that contains an additional adjuvant besides chitosan if desired to further modulate the response.

Moreover, Zhang *et al.* (2017) evaluated the safety profile of chitosan by comparing it with a commercially available adjuvant, ISA206, that causes the formation of microscopic lesions upon administration [113, 114]. For this purpose, they developed a chitosan-conjugated porcine circovirus type 2 (PCV2). In their studies, the chitosan-conjugated PCV2 vaccine was highly biocompatible. For example, no microscopic lesions were observed at the injection site of injected mice, unlike the PCV2 vaccine that contains the ISA206. Furthermore, the chitosan-PCV2 vaccine elicited a humoral and cellular immune response similar to PCV2/ISA206 [113, 114]. Avoiding tissue damage may reduce the inflammatory response associated with adverse effects such as pain and swelling.

Penetration of antigens into the NALT upon nasal administration is also a crucial factor to get high vaccine efficacy which in turn requires mucosal penetration. Size is negatively correlated to mucus penetration via diffusion through aqueous channels in the complex network of glycoproteins in mucus. Once penetrated through the mucus, smaller particles can diffuse through the mucosal membranes via the paracellular route, while larger particles need to go through transcellular transport via M-type epithelial cells [109, 115–117] with subsequent presentation of antigen to dendritic cells. Since TMC-OVA conjugates are considerably smaller structures (28 nm) in comparison to TMC nanoparticles (300 nm), they displayed higher penetration ability and better immunogenicity in comparison to the TMC nanoparticle vaccines [109]. Enhanced systemic (Th2 biased) and mucosal IgA immune responses were also observed when another chitosan derivative, N-trimethylaminoethylmethacrylate, was conjugated to OVA and tested intranasally [108]. Interestingly, TMC-OVA triggered a Th1-biased immune response, however, N-trimethylaminoethylmethacrylate-OVA triggered a Th2-biased immune response [108, 109]. Although this difference can be attributed to several parameters, such as dose and size, it is noteworthy that the type of chitosan derivative can also be a source of this difference. The route of uptake and intracellular processing of antigen or antigen in particles will affect the cytokine expression in dendritic cells, however, this is poorly understood in this specific context. This indicates a gap in the literature regarding chitosan conjugate vaccines and needs to be investigated further, as the application of chitosan conjugates as a vaccine formulation is a promising strategy.

Based on the result of these studies, conjugated forms of chitosan seem attractive alternatives to particulate carriers for vaccine delivery because of their greater mucosal penetration along with their potential to reduce the required antigen dose where a dose-sparing effect is accomplished. However, covalent binding to the antigen may inhibit antigen immunogenicity, and this has to be evaluated on a case-by-case basis, and consideration must be made so that the covalent linkage is biodegradable for antigen release.

## Chitosan as a coating material

Besides usage as the main component of vaccine delivery systems, chitosan has also been investigated as a coating material for other vaccine delivery systems such as nanoparticles and microparticles. The main reasons for using chitosan as a coating material are its mucoadhesivity and penetration enhancement ability. The development of mucosal vaccines, such as intranasal and oral vaccines, is a particular goal of those studies.

### Chitosan coating for PLGA cores

Among chitosan-coated delivery systems, PLGA micro- and nanoparticles are the most studied particulate vaccines as the core components. For instance, chitosan and its derivate TMC were used as coating material for hepatitis B surface antigen-loaded PLGA microparticles to optimize them for intranasal administration by increasing retention time in the nasal cavity. According to their results, TMC and chitosan coating significantly improved mucoadhesiveness and immunogenicity of the PLGA microparticles. TMC-coated particles were superior to native chitosan-coated vaccine formulations in terms of both mucoadhesiveness and immunogenicity [118]. Li *et al.* (2016) also used chitosan-coated PLGA microparticles for intranasal hepatitis vaccine delivery [119]. Their investigation showed a beneficial effect of chitosan coating on nasal retention, cellular disposition, cellular release, and immune response outcomes. Chitosan coating increased cellular uptake from 13.1% to 33.2% compared to uncoated nanoparticles. Antigen was released slowly from the chitosan-coated microparticles in the cytoplasm following cellular uptake, while uncoated particles released antigen quickly in endosomes and lysosomes of macrophages. This highlights the essential property of endosomal escape of chitosan-coated particles, which is necessary to avoid antigen degradation. Regarding intranasal retention of formulations, 54% of uncoated particles were retained in the nasal cavity after 10 min and a negligible amount after one hour. In contrast, 90% and 33% of chitosan-coated particles were retained in the nasal cavity after 10 min and one hour, respectively. Since chitosan coating increased both mucoadhesiveness and cellular uptake of microparticles, significantly higher humoral and cellular immune responses were also achieved with the coated formulations [119]. Similar results were also obtained when chitosan was used as a coating material for PLGA nanoparticles. For example, Pawar *et al.* (2013) tested chitosan and glycol chitosan-coated PLGA nanoparticles for intranasal hepatitis vaccine delivery and revealed that the magnitude of intranasal retention and uptake, systemic distribution, and immune response outcomes (humoral, cellular, local, and systemic) were in the order of glycol chitosan-PLGA > chitosan-PLGA > PLGA > antigen alone [120]. TMC was also tested in comparative studies as a coating material for PLGA nanoparticles. PLGA nanoparticles were coated with chitosan and TMC to deliver three *Mycobacterium tuberculosis* antigens together. The efficacy of those coatings was tested in two administration routes, subcutaneous and intranasal. Results showed that TMC coating is more efficient in terms of systemic immune response outcomes in comparison to chitosan-coated nanoparticles at with both routes of administration. Additionally, both TMC-PLGA and chitosan-PLGA nanoparticles evoked a significantly greater systemic immune response (TH1 biased) in comparison to uncoated PLGA nanoparticles upon both subcutaneous and nasal administrations [121]. Kaneko *et al.* (2021) used four different chitosan derivatives to coat antigen-loaded PLGA nanoparticles, and they evaluated the effect of the coating material on the dendritic activation that indicates essential for immunogenicity by analyzing the expression of cell surface markers, CD40 and CD86 [122]. The highest expression of both markers was obtained with chitosan hydrochloride, while glycol chitosan showed the lowest efficiency at inducing the expression of those markers. At this point, it is essential to note that these differences in the immunogenicity cannot only be attributed to the type of coating derivatives but also to other interacting particle features, such as size, surface charge, polydispersity index, and molecular weight of chitosan derivatives, and the antigen itself need to be considered [122].

### Chitosan coating for lipid-based cores

Chitosan has also been used to coat liposomal vaccine delivery systems. Using quaternized chitosan (QCS) coating for liposomes has been explored for the adjuvant resveratrol. Compared to non-coated liposomes, a 50-fold higher cellular uptake was observed with QCS-coated liposomes, which was attributed to the positive charge of QCS. Increased uptake of formulations resulted in co-localization of both antigen (OVA) and adjuvant in bone marrow dendritic cells which, as discussed above, is important for a desirable immune response. Besides its contribution as a coating material, it was also observed that chitosan could exhibit an adjuvant effect that enhanced immune response further [123]. In another study, TMC coating was used for two different cores: 1) alginate-coated liposomes loaded with lipopeptide antigens; and 2) alginate/lipopeptide antigen polyelectrolyte complex (PEC). Higher IgG and IgA titers were observed with PEC core upon the intranasal administration of the vaccine formulations. In that same study, the researchers also tested TMC as a coating for various PEC cores that consisted of different polymers (chondroitin sulfate, dextran, hyaluronic acid, and heparin) but the same lipopeptide antigen. Although the PECs had similar physicochemical properties, antigen payload, and surface coating, the different PECs showed different levels of immunogenicity which suggests that besides the coating material, the core material also needs to be considered carefully during the development of a core/shell type vaccine formulations [124]. This may be related, for instance, to the rate of coating dispersal, particle degradation rate, and antigen release post-administration.

### Chitosan coating for calcium phosphate-based cores

Chitosan-coated inorganic nanoparticles represent another class of vaccine delivery systems. Cao *et al.* (2020) used chitosan in two research studies as a coating material for calcium phosphate nanoparticles for oral vaccine delivery [125]. It was revealed that chitosan coating makes nanoparticles more stable in the gastrointestinal tract and increases their uptake in both dendritic and macrophage cell lines. Chitosan solubility is low at the pH of the stomach (∼pH 2–4); thus, coating with chitosan improves stability in the stomach. Researchers attributed the increased cellular uptake to the positive surface charge of chitosan and its binding ability to different surface receptors, such as mannose receptors on the macrophages [125]. In their second study, chitosan and *o*-carboxymethyl chitosan (CMC) were used for the coating shell, which enhanced both the systemic and the mucosal immune responses with both coating types [126]. As well, in the framework of that study, they carried out detailed experiments regarding mucin adsorption in which they showed that mucoadhesion is not only a function of electrostatic interactions but also hydrogen bonding and physical entanglement. This is supported by the fact that although the CMC-coated nanoparticles had a zeta potential of −4.7 mV, which should repel negatively charged mucous, they showed a similar degree of mucosal adhesion as chitosan-coated nanoparticles that had zeta potentials of +8.5 mV. In addition, they measured the diffusion rates of nanoparticles through mucus and observed that CMC-coated particles diffused through the mucus layer faster than chitosan-coated nanoparticles; this was attributed to the positive charge of chitosan-coated particles causing delay during mucosal penetration, whereas having a near-neutral charge eliminates the electrostatic interaction with mucus allowing for a faster diffusion rate. Transepithelial electrical resistance (TEER) assays were carried out to evaluate the impact of the particles on epithelial tight junction integrity, which affects the transit of the nanoparticles. Results showed that the increased permeation obtained with chitosan and CMC coating is due to the binding of the polymer shells to a transmembrane protein, JAM-1, which results in reversible disruption of tight junctions [126].

According to the aforementioned studies, chitosan coating positively contributes to mucoadhesiveness, nasal retention, mucosal penetration, cellular uptake, stability in the gastrointestinal tract, and immunogenicity of other vaccine delivery systems. Meanwhile, the comparative studies mentioned above also revealed that the magnitude of some of those positive contributions could depend on the type of chitosan (e.g., native or derivative) [118, 120]. However, there is a gap regarding this point, and further detailed studies are required to determine what type of contribution each derivative has on the properties of the final vaccine formulation.

Generally, since chitosan coating contributes to the improvement of multiple essential properties of delivery systems, it has been the subject of numerous studies, some of which are itemized in Table 2. It is also noteworthy that chitosan coating is also studied for the development of veterinary vaccines for diseases that are encountered among pigs [137, 138], tilapia [139, 140], chickens [141, 142], and other livestock [143–145].

**TABLE 2:** Vaccine delivery studies that used chitosan and its derivatives as coating material.

Dosage forms	Core material	Shell material	Payload	Administration route	Reference
Microparticle	PLGA	TMC	Hepatitis B surface antigen	Intranasal	[118]
Microparticle	PLGA	Chitosan	Hepatitis B surface antigen	Intranasal	[119]
Nanoparticle	PLGA	CMC, Chitosan HCl, Oligomers	OVA	N/A	[127]
Nanoparticle	PLGA	Chitosan Glycol Chitosan	Hepatitis B surface antigen	Intranasal	[120]
Nanosphere	PLGA	Chitosan, TMC	*Mycobacterium Tuberculosis* antigens	Intranasal Subcutaneous	[121]
Nanoparticle	PLGA	TMC, CMC, Chitosan HCl, Oligomer Chitosan Glutamate Glycol Chitosan	Pneumococcal surface protein A	N/A	[122]
Submicrometric particle	PLA	Chitosan	*Leishmania braziliensis* antigen	Intradermal	[128]
Nanoparticle	PCL	Chitosan	Influenza A virus H1N1 hemagglutinin protein	Intranasal	[129]
Nanoparticle	PGA-co-PDL	Chitosan HCl	BSA	N/A	[130]
Nanoparticle	DDAB TDB, PLGA	Glycol Chitosan	*Chlamydia trachomatis* fusion antigen	Intranasal	[131]
Nanoparticle	Liposome Soy phospholipids, cholesterol	QCS	OVA	Subcutaneous	[123]
Nanoparticle	Liposome DPPC DDAB	TMC	A synthetic β-sheet peptide (p3)	Oral	[132]
Nanoparticle	Liposome DPPC DDAB, Cholesterol	TMC	LCP-1	Intranasal	[124]
Nanoparticle	ISCOMATRIX	Chitosan	Human influenza (H1N1) virus (PR8)	Intramuscular, Intranasal	[133]
Nanoparticle	Bacille-Calmette-Guérin mycobacteria	Chitosan	Bacille-Calmette-Guérin mycobacteria	N/A	[134]
Nanoparticle	Bacteriophage	Chitosan	Bacteriophage	Intranasal	[135]
Microsphere	DNA hydrogel	Chitosan Glutamate	CpG ODN	Intraduodenal	[136]
Nanoparticle	Calcium phosphate	Chitosan	OVA, BSA	Oral	[125]
Nanoparticle	Calcium phosphate	Chitosan	OVA, BSA	Oral	[126]

Abbreviations: PLGA, poly(lactic-co-glycolic acid); TMC, trimethyl chitosan; CMC, carboxy-methyl chitosan; OVA, ovalbumin; PLA, poly(D,L-lactide); PCL, poly(ɛ-caprolactone); PGA-co-PDL, poly(glycerol adipate-co-ω-pentadecalactone); BSA, bovine serum albumin; DDAB, dimethyldiocta-decylammonium bromide; TDB, trehalose-6,6′-dibehenate; QCS, quaternized chitosan; DPPC, dipalmitoylphosphatidylcholine; LCP-1, lipopeptide; CpG ODN, cytosine-phosphate-guanine oligodeoxynucleotide.

## Discussion

Chitosan is a promising biomaterial for various vaccine applications. Its advantageous properties include its intrinsic immunogenicity, such that these systems can be self-adjuvanting. Moreover, it can also be used to enhance the immunogenicity of other adjuvants with complementary mechanisms of action, which may modulate the immunogenicity of the vaccine. Chitosan and its derivatives also enable the development of diverse delivery systems such as nanoparticles, microparticles, hydrogels, microneedles, and chemical conjugates. Each of these delivery systems may be modified to enable the loading of vaccine payloads in the form of whole viruses, subunit antigens, or nucleic acids. Their physicochemical properties can be designed to allow vaccine administration through different administration routes, such as intranasal, oral, intramuscular, and subcutaneous. Chitosan can also be used as a coating material to improve transfection efficiency, mucoadhesivity, and mucosal penetration. No commercially available vaccine formulation uses chitosan presently, however, with its versatile properties, chitosan has a great potential to be used in vaccine formulations in the future.

It should be noted that chitosan non-particulate delivery systems for vaccine delivery have not been extensively studied yet. In order to assess the effectiveness of each vaccine delivery system, it’s necessary to address their limitations. For instance, when developing chitosan-based hydrogel vaccines, it’s essential to consider the limitations of hydrogels such as delayed gelation, weak mechanical properties, high salt/gelation agent content, low cytocompatibility, and burst release [84–86]. As well, hydrogel payloads can alter the properties of hydrogels, including gelation time, temperature, pore structure, and mechanical properties. Therefore, the same hydrogel formulation can demonstrate different physiochemical characteristics with different vaccine payloads, which poses a challenge for optimization. Similarly, microneedles have certain limitations that can affect the efficacy of microneedle vaccine formulations. These limitations include dosage inaccuracy, limited loading capacity, tip breakage and/or clogging, and the need for multi-step preparation that can lead to optimization issues [146, 147]. Additionally, some vaccine payloads cannot be loaded in the microneedles due to their charge. The payload charge must be negative in order to be incorporated into the layers of the chitosan-coated microneedles [104, 105]. Meanwhile, as already mentioned above, conjugate vaccines also have limitations, mainly related to the reduction effect of conjugation on the activity of the payload antigen and off-target delivery that need to be explored and addressed.

In addition to addressing the limitations of delivery systems, there are other factors related to chitosan that need to be investigated as response variables during the experimental design of future non-particulate vaccine delivery studies. These factors include molecular weight, degree of deacetylation, type of derivatives, and the impact on the specific immune response.

In reviewing chitosan applications that are not as well-known as chitosan nanoparticles, we aimed to draw attention to the progress that has been made in those alternative approaches to vaccine delivery and to identify areas recommended for further investigation such as chitosan’s mechanisms of enhancing the immune response via its physical properties in a given formulation vs. its adjuvanticity by its direct stimulation of antigen-presenting cells.

## Figures and Tables

**Table:** 

**APC**	Antigen presenting cell
**BSA**	Bovine serum albumin
**cGAMP**	Cyclic-di-GMP-AMP
**cGAS**	Cyclic-di-GMP-AMP synthase
**CMC**	Carboxymethyl chitosan
**CpG ODN**	Cytosine-phosphate-guanine oligodeoxynucleotide
**CPMV**	Cowpea mosaic virus
**CS**	Chitosan
**DCs**	Dendritic cells
**DDAB**	Dimethyldiocta-decylammonium bromide
**DPPC**	Dipalmitoylphosphatidylcholine
**DT**	Diphtheria toxoid
**GM-CSF**	Granulocyte-macrophage colony-stimulating factor
**HBc**	Hepatitis B core protein
**HPMC**	Hydroxypropyl methylcellulose
**HTCC**	N-[(2-hydroxy-3-trimethylammonium) propyl] chitosan chloride
**IPV**	Inactivated polio vaccine
**LB**	Lipid bilayer
**LCP-1**	Lipopeptide
**LPS**	Lipopolysaccharide
**MSN**	Mesoporous silica nanoparticles
**mtDNA**	Mitochondrial DNA
**NALT**	Nasal-associated lymphoid tissue
**NPs**	Nanoparticles
**OVA**	Ovalbumin
**PCL**	Poly-(ɛ-caprolactone)
**PCS**	Phosphorylated chitosan
**PCV2**	Porcine circovirus type 2
**PEC**	Polyelectrolyte complex
**PGA-co-PDL**	Poly(glycerol adipate-co-ω-pentadecalactone)
**PLA**	Poly(L-lactide-co-D,L-lactide)
**PLGA**	Poly(lactic-co-glycolic acid)
**poly (I:C)**	Polyinosinic-polycytidylic acid
**PVA**	Polyvinyl alcohol
**PVP**	Polyvinyl pyrrolidone
**QCS**	Quaternized chitosan
**QD**	Quaternization degree
**rHBs**	Recombinant hepatitis B surface antigen
**SALT**	Skin-associated lymphoid tissues
**sIgA**	Secretion of mucosal secretory iga
**STING**	Stimulator of IFN genes
**TDB**	Trehalose-6,6′-dibehenate
**TMC**	Trimethyl chitosan
